# Association between pesticide exposure and obesity: A cross-sectional study of 20,295 farmers in Thailand

**DOI:** 10.12688/f1000research.53261.2

**Published:** 2022-02-02

**Authors:** Kajohnsak Noppakun, Chudchawal Juntarawijit

**Affiliations:** 1 Department of Internal Medicine, Faculty of Medicine, Chiang Mai University, Chiang Mai, Thailand; 2 Pharmacoepidemiology and Statistics Research Center (PESRC), Faculty of Pharmacy, Chiang Mai University, Chiang Mai, Thailand; 3 Faculty of Agriculture, Natural Resources and Environment, Naresuan University, Phitsanulok, 65000, Thailand

**Keywords:** Pesticide exposure, obesity, farmer health, insecticide exposure, herbicide exposure, fungicide exposure

## Abstract

**Background:** Obesity is a serious condition because it is associated with other chronic diseases which affect the quality of life. In addition to problems associated with diet and exercise, recent research has found that pesticide exposure might be another important risk factor. The objective of this study was to determine the association between pesticide exposure and obesity among farmers in Nakhon Sawan and Phitsanulok province, Thailand.

**Methods:** This study was a population-based cross-sectional study. Data on pesticide use and obesity prevalence from 20,295 farmers aged 20 years and older were collected using an in-person interview questionnaire. The association was analysed using multivariable logistic regression, adjusted for its potential confounding factors.

**Results:** Obesity was found to be associated with pesticide use in the past. The risk of obesity was significantly predicted by types of pesticides, including insecticides (OR = 2.27, 95% CI 1.09-4.74), herbicides (OR = 4.72, 95% CI 1.16-19.29), fungicides (OR = 2.17, 95% CI 1.37-3.44), rodenticides (OR = 2.52, 95% CI 1.59-3.99), and molluscicides (OR = 3.37, 95% CI 2.13-5.31). Among 35 surveyed individual pesticides, 24 were significantly associated with higher obesity prevalence (OR = 1.75, 95% CI 1.00-3.06 to OR = 8.37, 95% CI 3.97-17.64), including herbicide butachlor, 17 insecticides (three carbamate insecticides, five organochlorine insecticides, and nine organophosphate insecticides), and six fungicides.

**Conclusion:** This study found obesity in farmers in Nakhon Sawan and Phitsanulok province, Thailand, to be associated with the long-term use of several types of pesticides. The issue should receive more public attention, and pesticide use should be strictly controlled.

## Abbreviations

BMI: body mass index

CM: carbamate pesticide

CVD: cardiovascular diseases

2,5-DCP: 2,5-dichlorophenol

DDT: dichlorodiphenyltrichloroethane

EDC: endocrine-disrupting chemicals

ICD-10: International Classification of Diseases 10 ^th^

OC: organochlorine pesticide

OPs: organophosphate pesticide

PCBs: polychlorinated biphenyl

p,p'-DDE: dichlorodiphenyldichloroethylene

VHV: village health volunteers

## Background

Obesity is a global public health problem. In 2016, the World Health Organization (WHO) reported that there were approximately two billion people aged 18 years and older who were overweight, of which 650 million were obese, with this number expected to rise. ^1^ In Thailand, the latest national survey reported obesity prevalence among adults aged 18 years and older, to be 4.0% class I obesity (body mass index (BMI) 30.0-34.9 kg/m ^2^), 0.8% class II obesity (BMI 35.0-39.9 kg/m ^2^), and 0.1% class III obesity (BMI ≥40.0 kg/m ^2^). ^2^ Obesity is not just an image problem, it can also affect health and well-being. Obesity has been linked with various health problems, including cardiovascular disease (CVD), type 2 diabetes mellitus (T2DM), cancer, and other health problems including liver and kidney disease, sleep apnea, and depression, which can eventually lead to mortality. ^3^ Many factors can affect obesity, including age, genes, diet, a sedentary lifestyle, certain diseases, and medications, as well as other health conditions including sleeping habits, stress, and depression. ^4^

Recent studies have found that pesticide exposure may be associated with metabolic disorders such as obesity. ^5^ Mice studies have reported that chlorpyrifos, an organophosphate pesticide, has interfered with mucus-bacterial interactions in the gut, leading to increased lipopolysaccharide entry into the body resulting in excess fat storage. ^6^ A study in Korea found women with *Methanobacteriales, a* bacteria in the gut that is linked to obesity, have higher body weight and waist circumference. ^7^ This finding was consistent with a study in the United States of America (U.S.A) that with the use of the National Health and Nutrition Examination Survey (NHANES), found that obesity in adults was associated with the fumigant insecticide paradichlorobenzene, ^8^ and 2,5-dichlorophenol (2,5-DCP) exposure. ^9^ In a cohort study, dichlorodiphenyldichloroethylene (p,p'-DDE), and polychlorinated biphenyl (PCBs) were associated with higher BMI, high triglyceride levels, and insulin resistance. ^10^ Not only pesticides, a recent study also linked a simultaneous exposure to bisphenol A (BPA), bisphenol S (BPS), and mono (carboxyoctyl) phthalate (MCOP), to an elevated risk of obesity. ^11^

As far as we know, to date, this issue has not been investigated in Thailand. This cross-sectional study aimed to determine the association between pesticide exposure and obesity among farmers in Nakhon Sawan and Phitsanulok province, Thailand. The main interest was to associate obesity to pesticides, either by group or individual chemicals. The results will be useful for verification of previous results and prevention of obesity.

## Methods

### Study design and setting

Data in this cross-sectional study was collected from farmers in Nakhon Sawan and Phitsanulok province, Thailand. Nakhon Sawan province is located about 250 km north of Bangkok, Thailand, with a population of 1,066,455 people and 401,432 households, from 15 districts (data for the year 2016). The majority of people are farmers, and the main crops are rice, sugarcane, and cassava. In 2017, the province had a gross domestic product (GDP) of 21,852 THB (716 USD). ^12^ In 2019, Phitsanulok province, located 377 km north of Bangkok, had a population of 865,368 people from nine districts and 342,787 households. Agriculture is the biggest sector of the economy, generating about 28% of GDP with an employment rate of 41.9%. The major crops in the province are rice, sugar cane, cassava, and vegetables. ^13^^,^^14^

### Study participants and sampling procedure

Study participants were farmers aged 20 years and older, who had worked as farmers for at least five years. Participants were selected using multistage sampling. Firstly, three districts from each province were randomly selected. In each district, three sub-districts were further randomly selected. In each sub-district, we selected 2,100-4,500 farmer families, accounting for about 30-100% of all farmer families in each sub-district. Using data from the local authority and personal contact, village health volunteers (VHV) identified farmer families, who were contacted for data collection. In each family, one adult who met the inclusion criteria was interviewed.

All local hospitals inside the selected subdistrict were contacted and public health staff and its membership VHV were invited to participate in the study. VHV who had mobile phone and internet access to the online questionnaires were recruited. These volunteers also had to attend a one-day training session to be informed about the project and to be trained on interviewing the participants, along with the correct use of online questionnaires. Most of the interviews took place in the participant’s home, however sometimes in other places, e.g. local temple or hospital. Data was collected between October 2020 and February 2021. Data from all 20,295 participants were included in the data analysis.

The minimum sample size was calculated to be 18,772, based on the following assumptions: significance level = 95%; power of detection = 80%; ratio of unexposed/exposed = 1; percent of unexposed with outcome = 5%; odds ratio = 1.2. Exposed refers to exposure group, or those who used pesticides. Unexposed refers to unexposed group or those who do not use pesticides.

### Study questionnaire and data collection

Data was collected using an in-person interview questionnaire and an online version. The questionnaire had three major parts (provided as *Extended data* ^15^). Part I, contained demographic information, including gender, age, marital status and education. Information on cigarette smoking and alcohol consumption were also collected.

In part II, there were questions regarding the long-term use of pesticides. This question was modified from the questionnaire used in our previous study. ^16^ Data on both types and individual pesticides were collected. Pesticides were categorized into insecticides, herbicides, fungicides, rodenticides, and molluscicides. Insecticides were further subdivided into four classes: organochlorine, organophosphate, carbamate, and pyrethroid. For individual pesticides, we collected data on 35 pesticides that were commonly used in Thailand and have been reported in previous literatures to affect obesity. ^16^ Study participants were asked whether they have ever used the pesticides, using a ‘yes’ or ‘no’ question. Pesticide use was defined as a mixture or spray pesticides for agriculture purposes. Household pest control was excluded in this study.

In part III, participants were asked whether they had been medically diagnosed with obesity by using a “yes” or “no” question. This information was later confirmed by the disease record ICD-10 of the local hospitals. The confirmed cases were included in the data analysis, while the missing data was excluded. In Thailand, the Ministry of Public Health follows The World Health Organization obesity criteria for the Asia Pacific Region in which the body mass index over 25.0 kg/m ^2^ was identified as obesity. ^17^

### Statistical analysis

Demographic data were analysed using descriptive statistics (frequency, percentage, mean, and standard deviation). An association between pesticides and obesity was determined using multivariable logistic regression, adjusted for gender (male, female), age (20-30, 31-40, 41-50, 51-60, 60>), smoking (non-smoker, ex-smoker, smoker) and alcohol consumption (non-drinker (or abstainer), ex-drinker, regular drinker), presented in odds ratio (OR), 95% confidence intervals.

P-values <0.05 were statistically significant. All data analysis was performed using IBM SPSS Statistics (version 26) and OpenEpi online version 3.5.1.

## Ethical considerations

The study was approved by the Ethical Committee of Naresuan University (COA No. 657/2019). Before the interviews, all the study participants gave informed consent to participate in the study, and they had the right to stop the interview at any time.

## Results

Out of the 20,295 participants, 78 were medically diagnosed to be obese (0.4%) ( Table 1 and the *Underlying Data* ^18^). The case group also had other chronic diseases, e.g., hypertension (55.1%), diabetes mellitus (29.5%), and dyslipidaemia (14.1%). Only a small number of the cases currently smoke (7.7%) or drink alcohol (16.7%). Among other demographic characteristics, only age and cigarette smoking status were significantly associated with obesity (p <0.05).

**Table 1.** Characteristics of not obese, and obese participants.

	Not obese N (%)	Obese N (%)	P value **
Obesity	20217 (99.6)	78 (0.4)	
Having other related diseases			<0.001 *
-Hypertension	5402 (26.7)	43 (55.1)	
-Diabetes mellitus	2208 (10.9)	23 (29.5)	
-Dyslipidemia	1871 (9.3)	11 (14.1)	
-Heart disease	243 (1.2)	2 (2.6)	
-Sleep disorder	14 (0.1)	2 (2.6)	
-Stroke	331 (1.6)	1 (1.3)	
Gender			0.173
Male	9072 (44.9)	29 (37.2)	
Female	11145 (55.1)	49 (62.8)	
Age, yr			0.030 *
20-30	722 (3.6)	4 (5.1)	
31-40	1830 (9.1)	11 (14.1)	
41-50	4238 (21.0)	24 (30.8)	
51-60	6518 (32.2)	23 (29.5)	
61+	6909 (34.2)	16 (20.5)	
Mean ± SD = 55 ± 12			
Min-Max = 20-98			
District			<0.001 *
PH-WB	4518 (22.3)	0 (0)	
PH-BK	2106 (10.4)	25 (32.1)	
PH-BR	2991 (14.8)	9 (11.5)	
NS-LY	3364 (16.6)	15 (19.2)	
NS-MW	3662 (18.1)	9 (11.5)	
NS-TT	3576 (17.7)	20 (25.6)	
Marital status			0.657
Married	15476 (76.5)	63 (80.8)	
Single	1727 (8.5)	6 (7.7)	
Divorced/widowed/separated	3014 (14.9)	9 (11.5)	
Education level			<0.001 *
Not attend school	838 (4.1)	1 (1.3)	
Primary school	14915 (73.8)	40 (51.3)	
Secondary school	4158 (20.6)	32 (41.0)	
College degree or higher	306 (1.5)	5 (6.4)	
Income per month (THB)			0.021 *
<5000	7317 (36.2)	30 (38.5)	
5001-10000	10887 (63.9)	36 (46.2)	
10001-30000	1844 (9.1)	9 (11.5)	
>30000	169 (0.8)	3 (3.8)	
Cigarette smoke (n = 20217)			0.040 *
Non- smoke	17043 (84.3)	64 (82.1)	
Ex-smoker	918 (4.5)	8 (10.3)	
Current smoker	2256 (11.2)	6 (7.7)	
Alcohol consumption (n = 20217)			0.557
Non-drinker	15553 (76.9)	57 (73.1)	
Ex-drinker	1461 (7.2)	8 (10.3)	
Regular drinker	3203 (15.8)	13 (16.7)	

* Statistically significant difference with p-value <0.05.

** Chi-square test.

It was found that all types of pesticides, including insecticides, herbicides, fungicides, rodenticides, and molluscicides, were significantly associated with obesity prevalence ( Table 2). The associations were also found in many of the surveyed individual pesticides ( Table 3). Those pesticides were from various types of pesticide, including herbicides butachlor, 18 insecticides (three carbamate (CM) insecticide, five organochlorine pesticides (OC) insecticide, and nine organophosphate pesticides (OP) insecticide), and six fungicides.

**Table 2.** Association (OR) between different types of pesticide and obesity.

	Not obese (N = 20217) N (%)	Obese (N = 78) N (%)	OR (crude)	OR (adjusted) *
Any pesticide				
No	1092 (99.3)	8 (0.7)	1.0	1.0
Yes	19113 (99.6)	70 (0.4)	0.50 (0.24-1.04)	0.49 (0.23-1.02)
Insecticide				
No	4246 (99.8)	8 (0.2)	1.0	1.0
Yes	15959 (99.6)	70 (0.4)	2.33 (1.12-4.84) **	**2.27 (1.09-4.74)**
Herbicide				
No	2292 (99.9)	2 (0.1)	1.0	1.0
Yes	17913 (99.6)	76 (0.4)	**4.86 (1.19-19.81)**	**4.72 (1.16-19.29)**
Fungicide				
No	12071 (99.7)	31 (0.3)	1.0	1.0
Yes	8124 (99.4)	47 (0.6)	**2.25 (1.43-3.54)**	**2.17 (1.37-3.44)**
Rodenticide				
No	15800 (99.7)	46 (0.3)	1.0	1.0
Yes	4405 (99.3)	32 (0.7)	**2.50 (1.59-3.92)**	**2.52 (1.59-3.99)**
Molluscicide				
No	15357 (99.8)	38 (0.2)	1.0	1.0
Yes	4848 (99.2)	40 (0.8)	**3.33 (2.14-5.20)**	**3.37 (2.13-5.31)**

* Adjusted variables: Gender (male, female), age (20-30, 31-40, 41-50, 51-60, 60+), smoking (never, ex-smoker, current smoker), alcohol consumption (never, used to drink, currently drink).

** Significant OR were indicated in bold numbers.

**Table 3.** Association (OR) between individual pesticide and obesity.

Pesticide	Not obese (N = 20217) N (%)	Obese (N = 78) N (%)	OR crude	OR adjusted
**Herbicide**				
Glyphosate				
No	5715 (99.7)	17 (0.3)	1.0	1.0
Yes	14490 (99.6)	61 (0.4)	1.42 (0.83-2.43)	1.41 (0.82-2.42)
Paraquat				
No	10617 (99.7)	37 (0.3)	1.0	1.0
Yes	9588 (99.6)	41 (0.4)	1.23 (0.79-1.92)	1.20 (0.77-1.87)
D24				
No	10527 (99.7)	35 (0.3)	1.0	1.0
Yes	9678 (99.6)	43 (0.4)	1.34 (0.86-2.10)	1.32 (0.85-2.07)
Butachlor				
No	16025 (99.7)	43 (0.3)	1.0	1.0
Yes	4180 (99.2)	35 (0.8)	3.12 (2.00-4.88) **	**3.05 (1.94-4.79)**
Alachlor				
No	18660 (99.6)	68 (0.4)	1.0	1.0
Yes	1545 (99.4)	10 (0.6)	1.78 (0.91-3.46)	1.69 (0.87-3.30)
Diuron				
No	19798 (99.6)	74 (0.4)	1.0	1.0
Yes	407 (99.0)	4 (1.0)	2.63 (0.96-7.23)	2.48 (0.90-6.85)
**Organophosphate insecticide**				
Abamectin				
No	9570 (99.7)	25 (0.3)	1.0	1.0
Yes	10635 (99.5)	53 (0.5)	**1.91 (1.19-3.07)**	**1.87 (1.16-3.02)**
Chlorpyrifos				
No	15459 (99.7)	40 (0.3)	1.0	1.0
Yes	4746 (99.2)	38 (0.8)	***3.09 (1.98-4.83)***	***2.98 (1.90-4.68)***
Folidol (parathion)				
No	17665 (99.7)	62 (0.3)	1.0	1.0
Yes	2540 (99.4)	16 (0.6)	**1.80 (1.03-3.11)**	**1.75 (1.00-3.06)**
Methamidophos				
No	19430 (99.6)	71 (0.4)	1.0	1.0
Yes	775 (99.1)	7 (0.9)	**2.47 (1.13-5.39)**	**2.37 (1.08-5.19)**
Monocrotophos				
No	19743 (99.6)	72 (0.4)	1.0	1.0
Yes	462 (98.7)	6 (1.3)	**3.56 (1.54-8.23)**	**3.58 (1.55-8.39)**
Mevinphos				
No	19965 (99.6)	74 (0.4)	1.0	1.0
Yes	240 (98.4)	4 (1.6)	**4.50 (1.63-12.40)**	**4.35 (1.57-12.06)**
Dicrotophos				
No	19747 (99.6)	72 (0.4)	1.0	1.0
Yes	459 (98.7)	6 (1.3)	**3.59 (1.55-8.51)**	**3.51 (1.51-8.15)**
Dichlorvos				
No	19987 (99.6)	75 (0.4)	1.0	1.0
Yes	218 (98.6)	3 (1.4)	**3.67 (1.15-11.72)**	**3.74 (1.17-11.98)**
EPN				
No	19620 (99.6)	75 (0.4)	1.0	1.0
Yes	585 (99.5)	3 (0.5)	1.34 (0.42-4.27)	1.32 (0.41-4.22)
Imidacloprid				
No	19524 (99.6)	73 (0.4)	1.0	1.0
Yes	681 (99.3)	5 (0.7)	1.96 (0.79-4.88)	1.90 (0.76-4.72)
Profenofos				
No	19719 (99.6)	72 (0.4)	1.0	1.0
Yes	486 (98.8)	6 (1.2)	**3.38 (1.46-7.81)**	**3.15 (1.35-7.31)**
**Carbamate insecticide**				
Carbaryl				
No	18948 (99.7)	64 (0.3)	1.0	1.0
Yes	1257 (98.9)	14 (1.1)	**3.30 (1.84-5.90)**	**3.32 (1.85-5.96)**
Methomyl				
No	18879 (99.6)	68 (0.4)	1.0	1.0
Yes	1326 (99.3)	10 (0.7)	**2.09 (1.08-4.08)**	**1.99 (1.02-3.91)**
Carbosulfan				
No	17714 (99.7)	56 (0.3)	1.0	1.0
Yes	2491 (99.1)	22 (0.9)	**2.79 (1.70-4.58)**	**2.67 (1.62-4.40)**
Carbofuran				
No	18088 (99.6)	64 (0.4)	1.0	1.0
Yes	2117 (99.3)	14 (0.7)	1.87 (1.05-3.34)	1.75 (0.97-3.17)
**Pyrethroid insecticide**				
Permethrin				
No	17769 (99.6)	67 (0.4)	1.0	1.0
Yes	2436 (99.6)	11 (0.4)	1.20 (0.63-2.27)	1.14 (0.60-2.17)
**Organochlorine insecticide**				
Endosulfan				
No	17127 (99.7)	53 (0.3)	1.0	1.0
Yes	3078 (99.2)	25 (0.8)	**2.63 (1.63-4.23)**	**2.56 (1.58-4.18)**
Dieldrin				
No	20026 (99.6)	75 (0.4)	1.0	1.0
Yes	179 (98.4)	3 (1.6)	**8.92 (2.57-26.75)**	**8.18 (2.52-26.59)**
Aldrin				
No	20108 (99.6)	75 (0.4)	1.0	1.0
Yes	97 (97.0)	3 (3.0)	**8.29 (2.57-26.75)**	**8.18 (2.52-26.59)**
DDT				
No	19285 (99.6)	69 (0.4)	1.0	1.0
Yes	920 (99.0)	9 (1.0)	**2.73 (1.36-5.49)**	**2.74 (1.36-5.53)**
Chlordane				
No	19929 (99.6)	70 (0.4)	1.0	1.0
Yes	276 (97.2)	8 (2.8)	**8.25 (3.93-17.31)**	**8.37 (3.97-17.64)**
Heptachlor				
No	17046 (99.6)	62 (0.4)	1.0	1.0
Yes	3159 (99.5)	16 (0.5)	1.39 (0.80-2.42)	1.36 (0.79-2.37)
**Fungicide**				
Metalaxyl				
No	18638 (99.7)	63 (0.3)	1.0	1.0
Yes	1567 (99.1)	15 (0.9)	**2.83 (1.61-4.99)**	**2.68 (1.51-4.74)**
Mancozeb				
No	18843 (99.6)	70 (0.4)	1.0	1.0
Yes	1362 (99.4)	8 (0.6)	1.58 (0.76-3.29)	1.47 (0.70-3.08)
Maneb				
No	19280 (99.6)	71 (0.4)	1.0	1.0
Yes	92 5(99.2)	7 (0.8)	2.06 (0.94-4.48)	2.02 (0.92-4.41)
Propineb				
No	19261 (99.6)	68 (0.4)	1.0	1.0
Yes	944 (99.0)	10 (1.0)	**3.00 (1.54-5.85)**	**2.89 (1.48-5.64)**
carbendazim				
No	17904 (99.7)	57 (0.3)	1.0	1.0
Yes	2301 (99.1)	21 (0.9)	**2.87 (1.74-4.74)**	**2.71 (1.64-4.50)**
	<0.001 *			
Thiophanate				
No	19831 (99.6)	73 (0.4)	1.0	1.0
Yes	374 (98.7)	5 (1.3)	**3.63 (1.46-9.04)**	**3.52 (1.41-8.78)**
Benomyl				
No	19965 (99.6)	74 (0.4)	1.0	1.0
Yes	240 (98.4)	4 (1.6)	**4.50 (1.63-12.40)**	**4.58 (1.65-12.68)**
Bordeaux mixture				
No	20096 (99.6)	76 (0.4)	1.0	1.0
Yes	109 (98.2)	2 (1.8)	**4.85 (1.18-20.00)**	**5.35 (1.29-22.16)**

* Adjusted variables: Gender (male, female), age (20-30, 31-40, 41-50, 51-60, 60+), smoking (never, ex-smoker, current smoker), alcohol consumption (never, used to drink, currently drink).

** Significant OR were indicated in bold numbers.

## Discussion

In this study, the prevalence of obesity was very low (0.4%). This group of patients represent those with severe obesity or other health problems that needed medical attention. This was consistent with the data from a national survey in Thailand which found that the prevalence of obesity among adults aged 18 years and older to be 4.0% class I obesity (BMI 30.0-34.9 kg/m ^2^), 0.8% class II obesity (BMI 35.0-39.9 kg/m ^2^), and 0.1% class III obesity (BMI ≥40.0 kg/m ^2^). ^2^ In further analysis, this study also found that many of the obesity cases have other health problems (hypertension (55%), T2DM (30%)) ( Table 1).

This results of this study also found that many pesticides are strongly associated with the prevalence of obesity. The risk of obesity was significantly predicted by various types of pesticides, including insecticides (OR = 2.27, 95% CI 1.09-4.74), herbicides (OR = 4.72, 95% CI 1.16-19.29), fungicides (OR = 2.17, 95% CI 1.37-3.44), rodenticides (OR = 2.52, 95% CI 1.59-3.99), and molluscicides (OR = 3.37, 95% CI 2.13-5.31) ( Table 2). Among 35 surveyed individual pesticides 24 were significantly associated with obesity (OR = 1.75, 95% CI 1.00-3.06 to OR = 8.37, 95% CI 3.97-17.64), including herbicide butachlor, 17 insecticides (three CMs- carbaryl, methomyl, carbosulfan, five organochlorine insecticides- endosulfan, dieldrin, aldrin, DDT, chlordane, and nine organophosphate insecticides- abamectin, chlorpyrifos, folidol (parathion), methamidophos, monocrotophos, mevinphos, dicrotophos, dichlorvos, profenofos), and six fungicides- metalaxyl, propineb, carbendazim, thiophanate, benomyl, bordeaux mixture ( Table 3). Turnbaugh et al, ^19^ found pesticides affect the gut microbiome that controls the energy harvest, which may lead to obesity. This finding was supported by a recent study that found long-term exposure to chlorpyrifos affects gut microbiota homeostasis and induces inflammation, resulting in excess fat accumulation in the body. ^6^ Additionally, a Korean study reported on the Methanobacteriales in the gut being associated with increased waist circumference, and bodyweight. ^7^

Some pesticides are endocrine-disrupting chemicals (EDC). These are exogenous chemicals that interfere with the action of hormones, and/or obesogens, that inappropriately regulate lipid metabolism and adipogenesis to promote obesity. ^20^ At present, there are 105 pesticides listed as EDC, insecticides (46%) e.g. OCs DDT, 2,4-D, aldrin, endosulfan, chlorpyrifos, herbicide (21%) e.g. alachlor, diuron, glyphosate, and fungicides (31%) e.g., benomyl, carbendazim. A study found EDCs affect weight gain by altering lipid metabolism, fat cell size and number, and hormones involved in appetite, food preference, and energy metabolism. ^21^

Epidemiological studies on the association between pesticide exposure and obesity are rare. U.S.A National Health and Nutrition Examination Survey (NHANES) from 2005-2008, indicated that obesity of the general population was associated with environmental exposure to some pesticides, e.g. 2,4-dichlorophenol (2,4-DCP), and 2,5-dichlorophenol (2,5-DCP). ^8^ Among non-diabetic individuals, a study found that exposure to OC pesticides, especially p,p'-DDE, increased the risk of higher BMI, triglycerides, and decreased HDL cholesterol. ^10^ Another study using NHANES survey from 2003-2006, also found exposure to environmental pesticides increased obesity in children aged 6-19 years. ^22^ In this study, a dose-dependency was observed between the quartile of exposure to 2,5-DCP and the prevalence of obesity. These results were supported by a follow-up study which found 2,5-DCP exposure to be significantly associated with obesity (OR = 1.09, 95% CI 1.1-1.19) among children and adolescents aged 6-18 years. ^9^ A follow-up cohort study has found that middle-aged obese women were associated with mothers that used DDT, while pregnant with these women. (OR = 1.26, 95% CI 6-49 to OR = 1.31, 95% CI 6-62). ^23^

There were several limitations to the study that need to be mentioned. By using a cross-sectional design, the study was limited in explaining the causality since both disease and exposure data were examined at the same time. Though a large sample size was used, the number of obese participants was still small. This limited the power of detection and control of confounding factors. Data on other risk factors, such as diet, exercise, or genetics were not collected. These confounding factors might have a different impact on the results. However, the problem may not have much effect on the study results since the case and control groups were from the same community and having the same occupation. Small sample sizes also caused high values on the odds ratio with wide confidence intervals, therefore the result should be interpreted with caution. More study to confirm causal relationships between pesticide exposure and obesity was surely needed and the results should be used as a hypothesis generation. Another concern was that the obesity cases from ICD-10 records may not have been valid or represent the prevalence of the disease in the study. Currently, data on the validity of ICD-10 diagnosis coding for overweight/obesity in Thailand is not available. However, studies in Europe e.g., Sweden and Denmark, reported that the data is accurate and suitable to be used in epidemiological research. ^24^

In this study, information on pesticides exposure was solely based on the questionnaire method instead of biomarker or other individual quantitative measurement. This might cause concern over the information bias due to poor reliability and accuracy of the questionnaire data. However, for a large-scale study of long-term exposure to pesticides, this method may be the only option. Measurement of a biomarker in blood or urine is costly and may only represent short-term exposure. ^25^ For long-term exposure, using a questionnaire collecting data on the duration and intensity of pesticide use was accepted and has been used in many large-scale studies under the Agricultural Health Study in the United State for other diseases. ^26^ Also, by using a large number of individual pesticides to predict risk of obesity, it is very likely that the problem of co-exposure or the joint effect of mixed exposure to pesticides will occur. ^11^ This could distort the study result. Further study should try to do a dose-response analysis and study the effect of multiple pesticide exposure.

## Conclusion

In Nakhon Sawan and Phitsanulok province of Thailand, obesity in farmers was associated with the long-term use of several types of pesticides, including insecticides, herbicides, fungicides, rodenticides, and molluscicides. The study additionally found 24 individual pesticides was significantly associated with obesity. This finding was consistent with the literature and studies done in other countries. The information should be publicized, and pesticide use should be controlled. Further studies should be done to confirm the results, and to determine a safe limit of pesticide exposure for obesity risk.

## Data availability

### Underlying data

Figshare: Dataset for study on pesticide exposure and obesity, among farmers in Nakhon Sawan and Phitsanulok province, Thailand.

https://doi.org/10.6084/m9.figshare.14524983.v1. ^18^

This project contains the following underlying data:

• Dataset Pesticide and obesity (SAV and CSV). (All underlying data gathered in this study)
• Data Dictionary (DOCX).

### Extended data

Figshare: Dataset for study on pesticide exposure and obesity, among farmers in Nakhon Sawan and Phitsanulok province, Thailand.

https://doi.org/10.6084/m9.figshare.14524980.v1. ^15^

This project contains the following extended data:

• Questionnaire-pesticide and obesity-English (DOCX). (Study questionnaire in English)
• Questionnaire-pesticide and obesity-Thai (DOCX). (Study questionnaire in Thai)

Data are available under the terms of the Creative Commons Zero “No rights reserved” data waiver (CC0 1.0 Public domain dedication).
