# Supplementary material for: Association between pesticide exposure and obesity: A cross-sectional study of 20,295 farmers in Thailand
Source: F1000Res. 2022 May 17;10:445. Originally published 2021 Jun 4. [Version 3] doi: 10.12688/f1000research.53261.3 (PMC9127373; doi:10.12688/f1000research.53261.3)
Supplement: Peer review history

# Peer review: Reviewer response for version 3

**DOI:** 10.5256/f1000research.134032.r138054

**Published:** 2022-05-23

**Authors:** Erik Jors

**Affiliations:** 1 Clinic of Occupational Medicine, Clinical Institute, University of Southern Denmark, Odense, Denmark

I find that the paper with the changes in this third version can be indexed.

Is the work clearly and accurately presented and does it cite the current literature?

Partly

If applicable, is the statistical analysis and its interpretation appropriate?

Partly

Are all the source data underlying the results available to ensure full reproducibility?

Yes

Is the study design appropriate and is the work technically sound?

Partly

Are the conclusions drawn adequately supported by the results?

Partly

Are sufficient details of methods and analysis provided to allow replication by others?

Yes

Reviewer Expertise:

Pesticide poisonings

I confirm that I have read this submission and believe that I have an appropriate level of expertise to confirm that it is of an acceptable scientific standard.

# Peer review: Reviewer response for version 2

**DOI:** 10.5256/f1000research.79778.r128600

**Published:** 2022-04-07

**Authors:** Erik Jors

**Affiliations:** 1 Clinic of Occupational Medicine, Clinical Institute, University of Southern Denmark, Odense, Denmark

Dear Authors and Editors,

 I have read this interesting article on pesticide exposure and obesity among Thailand farmers. The research describes the association between obesity and the use of pesticides among farmers. It shows that there might be an association, but results have to be further analyzed before indexing, especially for relevant confounders like education and income and if possible for amounts of pesticides used. I, therefore, cannot approve the article for indexing but urge the authors to improve it before indexing can be reconsidered.

 I have the following comments and suggestions for revision:

 Major corrections:

1. In your analysis, you control for sex, age, smoking, and alcohol consumption which is fine, but you miss some important confounders of which you have data presented in Table 1 - income and educational level. Both might influence the use of pesticides and/or obesity, so I suggest you take them into your multivariate analysis.
2. You should have included the amounts and/or frequency of pesticide use. As it is now, one spraying per year counts equal to 100 sprayings per year, or 100 grams per year equals 1 kg per year. Please include this in your limitations if you do not have the data to include in your analysis as it is a major shortcoming.
3. Obesity often runs in families, you have no data on this but it should be discussed in the article.
4. In Table 2 you present results I do not understand - please discuss why the 'use of any pesticide' seems to be protective against obesity while the 'use of each of the specific pesticides' seems to be associated with obesity. This has to be discussed and explained.

Minor corrections:

1. The literature included could have been more comprehensive, please try to include more of the relevant literature.
2. In Tables 2-3, it would have been more illustrative to have the percentages among the not obese, like xx% uses pesticides, yy% do not, and the same for the obese.
3. It is interesting why this group of farmers has so less obesity than the general population - this must be discussed in the article.

When these issues are addressed I will be happy to review the article again.

Is the work clearly and accurately presented and does it cite the current literature?

Partly

If applicable, is the statistical analysis and its interpretation appropriate?

Partly

Are all the source data underlying the results available to ensure full reproducibility?

Yes

Is the study design appropriate and is the work technically sound?

Partly

Are the conclusions drawn adequately supported by the results?

Partly

Are sufficient details of methods and analysis provided to allow replication by others?

Yes

Reviewer Expertise:

Pesticide poisonings

I confirm that I have read this submission and believe that I have an appropriate level of expertise to confirm that it is of an acceptable scientific standard, however I have significant reservations, as outlined above.

# Response

**Published:** 2022-05-13

**Authors:** Chudchawal Juntarawijit

**Affiliations:** Naresuan University, Thailand

**Comment and response**

**Comment**

 I have read this interesting article on pesticide exposure and obesity among Thailand farmers. The research describes the association between obesity and the use of pesticides among farmers. It shows that there might be an association, but results have to be further analyzed before indexing, especially for relevant confounders like education and income and if possible for amounts of pesticides used. I, therefore, cannot approve the article for indexing but urge the authors to improve it before indexing can be reconsidered.

**Response**

 We would like to thank you so much for your time and effort in reviewing our paper. Your comments are very useful in helping to improve the quality of the paper.

**Major corrections:**

**Comment**

 In your analysis, you control for sex, age, smoking, and alcohol consumption which is fine, but you miss some important confounders of which you have data presented in Table 1 - income and educational level. Both might influence the use of pesticides and/or obesity, so I suggest you take them into your multivariate analysis.

**Response**

 As suggested, the data has been reanalyzed with two variables (income and education levels) added to the model. However, only minor changes to the OR were observed, but the overall levels and direction of the associations remain intact. The data in Tables 2-3 and in other related texts have been updated.

**Comment**

 You should have included the amounts and/or frequency of pesticide use. As it is now, one spraying per year counts equal to 100 sprayings per year, or 100 grams per year equals 1 kg per year. Please include this in your limitations if you do not have the data to include in your analysis as it is a major shortcoming.

**Response**

 Yes, we agree that the major limitation of this study was exposure assessment, but it might be the best available method for this type of study. The problem was also raised by other reviewers and the issue was already discussed and presented in the last paragraph of the discussion. Please see also the response to reviewer #1 and reviewer #2.

**Comment**

 Obesity often runs in families, you have no data on this but it should be discussed in the article.

**Response**

 To be a real confounding factor, the family history must be associated with both obesity and the use of pesticides. In this case, the family’s histories are strongly linked to obesity, but may not be to the use of pesticides. Due to their health, obese people tended to avoid using chemicals whenever possible. Therefore, family history was unlikely to affect the observed association of our study.

**Comment**

 In Table 2 you present results I do not understand - please discuss why the 'use of any pesticide' seems to be protective against obesity while the 'use of each of the specific pesticides' seems to be associated with obesity. This has to be discussed and explained.

**Response**

 The problem may come from the fact that there were many individual pesticides, while only some of them were associated with obesity. Therefore, the overall effect of any pesticides may or may not be the same as those of individual ones. In this study, only 22 out of the 35 individual pesticides were found to be significantly associated with obesity. In fact, there are more than 35 individual pesticides used in Thailand, and the term “pesticides” may be confusing to the participants. In addition, the data were from two different questions, the first was “have you ever used any pesticides?”, and the other one was “have you ever used, for instance, glyphosate?

 The problem may also stem from the inaccuracy of the exposure assessment of the chemicals when the questionnaire method is used. The issue has already been discussed and presented in the last paragraph of the Discussion.

**Minor corrections:**

**Comment**

 The literature included could have been more comprehensive, please try to include more of the relevant literature.

**Response**

 This issue is rather new, and so far there have only been a few studies on it carried out. To the best of our knowledge, all the relevant studies have already been reviewed.

**Comment**

 In Tables 2-3, it would have been more illustrative to have the percentages among the not obese, like xx% uses pesticides, yy% do not, and the same for the obese.

**Response**

 In Tables 2-3, the data on the use of pesticides among the obese and non-obese groups have been edited as you suggested. Thank you very much for your insightful advice.

**Comment**

 It is interesting why this group of farmers has so less obesity than the general population - this must be discussed in the article.

**Response**

 Although the prevalence rate of obesity among the study group (0.4%) was lower than that of the general population (4.0%), which referred to those with class I to class III obesity, if we consider 0.8% for class II, and 0.1% for class III, the prevalence was similar to those among the study group. In this study, the obese group is those who went to see the doctor and were registered in the ICD-10 of the hospital. Therefore, this group was more likely to suffer from severe obesity.

 This issue has already been mentioned in the first paragraph of the discussion. Sorry for the confusion, and to make it clearer, the paragraph has been revised.      

# Peer review: Reviewer response for version 2

**DOI:** 10.5256/f1000research.79778.r122025

**Published:** 2022-02-07

**Authors:** Yankai Xia

**Affiliations:** 1 State Key Laboratory of Reproductive Medicine, Center for Global Health, School of Public Health, Nanjing Medical University, Nanjing, China

The authors addressed most of my comments and I recommend acceptance.

Is the work clearly and accurately presented and does it cite the current literature?

Partly

If applicable, is the statistical analysis and its interpretation appropriate?

Yes

Are all the source data underlying the results available to ensure full reproducibility?

Yes

Is the study design appropriate and is the work technically sound?

Partly

Are the conclusions drawn adequately supported by the results?

Yes

Are sufficient details of methods and analysis provided to allow replication by others?

Yes

Reviewer Expertise:

Exposure to pesticides and health effects

I confirm that I have read this submission and believe that I have an appropriate level of expertise to confirm that it is of an acceptable scientific standard.

# Peer review: Reviewer response for version 1

**DOI:** 10.5256/f1000research.56626.r95935

**Published:** 2021-10-15

**Authors:** Yuki Ito

**Affiliations:** 1 Department of Occupational and Environmental Health, Nagoya City University Graduate School of Medical Sciences, Nagoya, Japan

The authors have investigated the relationships of pesticides used by farmers and obesity following the Thailand Ministry of Public Health definitions. They found a positive association between pesticide use and obesity. However, there are major points to be concerned as mentioned below. Also, the paper may benefit from an English language edit.

**Abstract:**

- Background in the Abstract should be specified by demonstrating the relationships between pesticides exposure and obesity.
- The authors should write the objective in the background section, not in the method section.

**Background:**

- The background should address the rationale of the study.
- In the last line of the first paragraph of the background section, the authors have stated lots of factors associated with obesity. Unfortunately, they cited only one article. More corresponding references should be added in this section.
- In the second paragraph, the authors described the association of PCBs, bisphenols, and one phthalate with obesity. Instead of these descriptions, the authors should cite other epidemiological studies examining the relationship between pesticide exposure and obesity.
- Authors stated that “identifications of individual pesticides to predict the risk of obesity was the main interest”. However, they did not assess the risk of pesticide exposure. They only investigated the observational relationships between the groups of pesticide use and obesity. The last line has no basis to write in this section.

**Methods:**

- The authors should remove the duplicate statement such as, “the major crops in the province are rice, sugar cane, casava and vegetables” from the study settings and design section.
- Please clarify the last two lines of “study participants and sampling procedure”. Define the term exposed and unexposed.
- A major concern is the definition of obesity. The authors investigated the relationship between pesticide use and obesity following the definition of the Thailand Ministry of Public Health. However, the definition of obesity is not clear. Based on the author’s statement, it seems like the definition of a metabolic syndrome but not obesity (see the article by Engin, 2017 ^1^). The authors should clarify it. They can consider BMI, WC, and/or other anthropometric indices of overweight and obesity if data is available.

**Results:**

- Out of 20,295, only 78 study participants were obese. The overall prevalence of obesity was very low, although the authors did not calculate prevalence both in males and females.
- Tables 1 and 2 were not prepared carefully. These two tables should merge and put data in each column and row carefully.
- If possible, please provide all the demographic data (collected by questionnaires), including socio-economic status, education, ethnicity, etc.
- For investigating the OR, they have adjusted the results only by gender, age, smoking, and alcohol consumption. They have no data about diet, physical activity, socioeconomic status or income that may affect the results.

**Discussion:**

- This section should be written based on results deeply and sharply. They should explain their findings, e.g. why and how by comparing other studies.
- Additionally, this study is only based on questionnaire data and did not monitor the individual exposure levels of pesticides. This is another major limitation of this study. 

**Conclusion:**

- The conclusion should be short and specific based on the results.
- The authors should not use “increased the risk of obesity”.

Is the work clearly and accurately presented and does it cite the current literature?

Partly

If applicable, is the statistical analysis and its interpretation appropriate?

Partly

Are all the source data underlying the results available to ensure full reproducibility?

Yes

Is the study design appropriate and is the work technically sound?

No

Are the conclusions drawn adequately supported by the results?

Partly

Are sufficient details of methods and analysis provided to allow replication by others?

Partly

Reviewer Expertise:

Exposure assessment of pesticides and their association with health effects.

I confirm that I have read this submission and believe that I have an appropriate level of expertise to state that I do not consider it to be of an acceptable scientific standard, for reasons outlined above.

## References

[1] : The Definition and Prevalence of Obesity and Metabolic Syndrome. *Adv Exp Med Biol* .2017;960: 10.1007/978-3-319-48382-5_1 1-17 10.1007/978-3-319-48382-5_1 28585193

# Response

**Published:** 2021-11-24

**Authors:** Chudchawal Juntarawijit

**Affiliations:** Naresuan University, Thailand

**Comment**

 The authors have investigated the relationships of pesticides used by farmers and obesity following the Thailand Ministry of Public Health definitions. They found a positive association between pesticide use and obesity. However, there are major points to be concerned as mentioned below. Also, the paper may benefit from an English language edit.

**Response**

 Thank you, we really appreciate your time and effort to review the manuscript.

 We admitted that this study may be far from perfect. By using a descriptive cross-sectional study, this study has several limitations. As compared to an analytical cross-sectional study, a better study design with a control group is often found in medical studies. With the descriptive design, this study had some limitations and could not be compared with the analytical one. This study lacks data on some confounding variables and relies only on the questionnaire method for pesticide exposure data. However, the results supported the usefulness of ICD-10 data for studying obesity. We hope that the result could be useful as a small piece of information to the relatively rare literature on the association of pesticides and obesity.

 The English language was edited by Mr. Kevin.

**Abstract:**

**Comment**

 Background in the Abstract should be specified by demonstrating the relationships between pesticides exposure and obesity.

**Response**

 Currently, there was not enough evidence demonstrating the relationship between pesticides exposure and obesity. With 250 words as the maximum word count limited by F1000Research, the issue, therefore, was only briefly described here.

**Comment**

 The authors should write the objective in the background section, not in the method section.

**Response**

 We agree and the objective has been revised and moved to the background section, as follows:

*Background: ...The objective of this study was to determine the association between pesticide exposure and obesity among farmers in Nakhon Sawan and Phitsanulok province, Thailand.*

*Methods: This study was a population-based cross-sectional study.*

**Background:**

**Comment**

 The background should address the rationale of the study.

**Response**

 The main reason why this study is needed was that obesity is an important public health problem and exposure to pesticides might be one of its causes. However, currently, there was only limited evidence. This study was among a few studies trying to investigate the association between obesity and pesticide exposure using a large cross-sectional study design.

**Comment**

 In the last line of the first paragraph of the background section, the authors have stated lots of factors associated with obesity. Unfortunately, they cited only one article. More corresponding references should be added in this section.

****

**Response**

 The reference was a systematic review that mentioned various factors associated with obesity.

**Comment**

 In the second paragraph, the authors described the association of PCBs, bisphenols, and one phthalate with obesity. Instead of these descriptions, the authors should cite other epidemiological studies examining the relationship between pesticide exposure and obesity.

****

**Response**

 In this section, we try to present information on pesticides and obesity. To our knowledge, all of the epidemiological studies on pesticides and obesity were already included. In the last statement, we just want to mention that not only pesticides but also some other compounds, e.g., PCBs, bisphenols, and one phthalate might also cause obesity.

 To make it clear, the statement has been revised.

**Comment**

 Authors stated that “identifications of individual pesticides to predict the risk of obesity was the main interest”. However, they did not assess the risk of pesticide exposure. They only investigated the observational relationships between the groups of pesticide use and obesity. The last line has no basis to write in this section.

**Response**

 To be more precise, the statement “ *identifications of individual pesticides to predict the risk of obesity was the main interest*” has been revised as follows:

*"The main interest was to associate obesity to pesticides, either by group or individual chemical.”*

**Methods:**

**Comment**

 The authors should remove the duplicate statement such as, “the major crops in the province are rice, sugar cane, cassava and vegetables” from the study settings and design section.

**Response**

 Actually, that statement was not duplicated but they described major crops of the two different provinces, Nakhon Sawan and Phitsanulok.

**Comment**

 Please clarify the last two lines of “study participants and sampling procedure”. Define the term exposed and unexposed.

****

**Response**

 Exposed refers to the exposure group or those who used pesticides. Unexposed refers to the unexposed group or those who do not use pesticides.

 This information has been added to the paper.

**Comment**

 A major concern is the definition of obesity. The authors investigated the relationship between pesticide use and obesity following the definition of the Thailand Ministry of Public Health. However, the definition of obesity is not clear. Based on the author’s statement, it seems like the definition of a metabolic syndrome but not obesity (see the article by Engin, 2017 ^1^ ). The authors should clarify it. They can consider BMI, WC, and/or other anthropometric indices of overweight and obesity if data is available.

**Response**

 We thank you the reviewer for making a very important point and agreeing to revise our manuscript as we picked up the case with obesity using a diagnosis in ICD-10. We have deleted the statement “ *In Thailand, the Ministry of Public Health follows the International Diabetes Federation’s definition (2005), which defines obesity as a waist circumference of no more than 90 cm for men and 80 cm for women, plus two out of the following four criteria:..*” and replaced with the following, “ *In Thailand, the Ministry of Public Health follows The World Health Organization obesity criteria for Asia Pacific Region in which body mass index over 25.0 kg/m2 was identified as obesity.".*

**Results:**

**Comment**

 Out of 20,295, only 78 study participants were obese. The overall prevalence of obesity was very low, although the authors did not calculate prevalence both in males and females.

****

**Response**

 Prevalence of obesity was very low since the study use data from ICD-10 records, instead of survey data on BMI or waist circumstance. The issue was already discussed in the manuscript.

**Comment**

 Tables 1 and 2 were not prepared carefully. These two tables should merge and put data in each column and row carefully.

****

**Response**

 The two tables have been merged. An error in the table head has been corrected.

**Comment**

 If possible, please provide all the demographic data (collected by questionnaires), including socioeconomic status, education, ethnicity, etc.

**Response**

 In this survey, we collected data from respondents who are farmers living in a rural area of six districts but found obesity cases in five districts (Table 1).

 For socioeconomic data, only the district location of participants, and their education, and monthly income were collected.  However, these factors were not included in the model because of several reasons. For district location, most of the participants were farmers from rural areas, thus there was no reason to believe district location to associate with obesity. Moreover, the district was closely related to pesticide use and will cause multicollinearity problems, if included in the model (Bhandari, 2020). For income, there was no relationship with obesity in middle-income countries, e.g. Thailand (Ameye and Swinnen, 2019).  For education, previous research found a higher risk of obesity to be inversely associated with education (Anyanwu, 2010). However, in this study more obesity was found among those with higher education (Table 1). In addition, when these two variables were included in the model, not much effects on OR were found. For example, for molluscicide the OR was changed from 2.28 (1.48-3.64) to 2.37 (1.48-3.79) when added education and to 2.39 (1.49-3.83) when added income (Table 2-1). For baseline diseases, most of them should be considered as the so-called ‘downstream outcome’ closely related to obesity and thus, should not be included in the model (DVL, 2020).

 More demographic information has been added to Table 1.

 For ethnicity, in Thailand, we did not have ethnic groups.

**Comment**

 For investigating the OR, they have adjusted the results only by gender, age, smoking, and alcohol consumption. They have no data about diet, physical activity, socioeconomic status or income that may affect the results.

**Response**

 Yes, we agree that that information on diet and physical activity would be useful and should be used for the adjustment. It is a study limitation and the issue was already discussed in the study limitation. The problem should not seriously affect study results since all of the participants were farmers from rural areas.

 For data on socioeconomic, actually, we also collected data on district location, education, and monthly income.  However, these factors were not included in the model because of several reasons. For district location, most of the participants were farmers from rural areas, thus there was no reason to believe district location to associate with obesity. However, the district was closely related to pesticide use and caused a multicollinearity problem, therefore, it was not be included in the model (Bhandari, 2020). For education and income, previous research found a higher risk of obesity to inversely associate with education (Anyanwu, 2010). For income, there was no relationship with obesity in middle-income countries, e.g. Thailand (Ameye and Swinnen, 2019).  When the model included these two variables, the OR was not much changed. For example, for Molluscicide the OR was changed from 2.28 (1.48-3.64) to 2.37 (1.48-3.79) when added education and to 2.39 (1.49-3.83) when added income (Table 2-1). For baseline diseases, most of them should be considered as the so called ‘downstream outcome’ closely related to obesity but not confounding and thus, should not be included in the model as well (DVL, 2020).

**Discussion:**

**Comment**

 This section should be written based on results deeply and sharply. They should explain their findings, e.g. why and how by comparing other studies.

**Response**

 Thank you for the suggestion. We agree and try to comply with your suggestion as much as we can.

**Comment**

 Additionally, this study is only based on questionnaire data and did not monitor the individual exposure levels of pesticides. This is another major limitation of this study. 

**Response**

 For a large study of long-term effects of pesticides, a questionnaire might be the only option. Since pesticide dose is a good marker for short-term pesticide exposure. However, it was costly and may not be suitable for a large survey study. The questionnaire method is probably the best alternative to collect data on long-term pesticide exposure. The method is commonly used in epidemiological studies e.g., Agricultural Health Study in the United State. For studies using a large group of participants, the method has been proved to be useful without any serious bias (Coble *et al*., 2011) (Hoppin *et al*., 2002).

 The limitation was further discussed as suggested.

**Conclusion:**

**Comment**

 The conclusion should be short and specific based on the results.

****

**Response**

 Thank you for the suggestion. We agree and try to comply with your suggestion as much as we can.

**Comment**

 The authors should not use “increased the risk of obesity”.

**Response**

 The statement has been changed to “ *The study additionally found exposure to 24 individual pesticides was significantly associated with obesity*."

**References**

 Anyanwu, G. E., Ekezie, J., Danborno, B., & Ugochukwu, A. I. (2010). Impact of education on obesity and blood pressure in developing countries: A study on the Ibos of Nigeria. North American Journal of Medical Sciences, 2(7), 320–324. https://doi.org/10.4297/najms.2010.2320

 Bhandari A. (2020). What is multicollinearity? Here’s everything you need to know. Analytics Vidhya. Available: Multicollinearity | Detecting Multicollinearity with VIF (analyticsvidhya.com)

 Coble *et al*., (2011). An updated algorithm for estimation of pesticide exposure intensity in the agricultural health study, Int. J. Environ. Res. Public Health; 8(12), 4608–4622.  doi: 10.3390/ijerph8124608.

 Hannah Ameye and Johan Swinnen, (2019). Obesity, income and gender: The changing global relationship, Global Food Security, 23, 267-281. https://doi.org/10.1016/j.gfs.2019.09.003.

 Hoppin, F. Yucel, M. Dosemeci, and D. P. Sandler, (2002). Accuracy of self-reported pesticide use duration information from licensed pesticide applicators in the Agricultural Health Study, J. Expo. Anal. Environ. Epidemiol.; 12(5), 313–318.  doi: 10.1038/sj.jea.7500232.

**Table 2-1 Odds ratio when more variables were added to the model.**

 Pesticide: Molluscicide

 OR (in the table 2)  3.36 (2.13-5.31)

 OR (when add baseline disease)   3.30 (2.08-5.22)

 OR (add district location)   2.28 (1.43-3.64)

 OR (add education)   2.37 (1.48-3.79)

 OR (add income)  2.39 (1.49-3.83)

# Peer review: Reviewer response for version 1

**DOI:** 10.5256/f1000research.56626.r94404

**Published:** 2021-10-01

**Authors:** Yankai Xia

**Affiliations:** 1 State Key Laboratory of Reproductive Medicine, Center for Global Health, School of Public Health, Nanjing Medical University, Nanjing, China

This study used questionnaire-based pesticide exposure data of 20,295 farmers to determine the association between pesticide use and obesity in Thailand. With the large-scale cross-sectional epidemiological data, the study determined that regional obesity in farmers could be associated with pesticide use. The study displayed novelty and value in public health on Thailand regional health. However, I thought there were several questions in this manuscript. Therefore, I thought major revision was necessary. The detailed review points were listed below.

1. In the abstract, the authors declared that they collected the long-term pesticide exposure data. However, I thought the term ‘long-term’ should be considered carefully as the questionnaire did not collect the frequencies of pesticides use (only yes or no). Also, the nature of the cross-sectional study (without follow-up) could poorly support the conclusion.
2. In the farmer population, pesticide-attributed obesity may be associated with the living conditions or geographical location of these individuals. Therefore, I thought it would be helpful if the authors provided the related data of these individuals.
3. Methods: Since there is a great difference between genders on obesity diagnosis, I recommended the authors perform stratified analysis to determine the gender-specific associations.
4. Results: The authors checked the relations between pesticide exposure and obesity. Is there any interaction between the different pesticides?
5. Results: The authors should clarify the demographic characteristics in detail.
6. Results: It will be helpful to display the C-index of each model to validate the reliability.
7. Discussion: I noticed that the OR for pesticides is from 1.75 to 8.37, while the OR over 3 may be overestimated and lead to unnecessary panic for public health. I thought the authors should discuss their results more carefully.
8. Discussion: I thought the authors should declare the nature of the cross-sectional study and tell the readers that the study was limited in explaining the causality.
9. Discussion: The strengthen part only replicates the conclusion.
10. One of the most limited parts of this study was the lack of dose or level data of pesticides use, which may cause serious bias. The authors should make a discussion about this.
11. The authors should explain the reliability and validity of their questionnaire as there may be information bias that exists with the questionnaire method.
12. I recommended the authors list the data on body measure index or waist circumstances in Table 1.
13. Waist circumferences and baseline diseases should be adjusted as covariates.
14. According to the previous literature, there is a great difference between populations in different age groups. Therefore, I thought age-specific associations between pesticides use and obesity should be considered.
15. As the authors declared, the limited data on dietary patterns, physical activities, and genetic variants may lead to potential bias. However, I thought the authors should be concerned about whether their adjusted models could explain the variance.
16. Could the authors list the blood pressure, glucose, triglyceride, and HDL value in the tables? Also, I think adjusting them in the model should be considered.
17. The effects of pesticides exposure may have co-exposure patterns, the authors should notice that.

Is the work clearly and accurately presented and does it cite the current literature?

Partly

If applicable, is the statistical analysis and its interpretation appropriate?

Yes

Are all the source data underlying the results available to ensure full reproducibility?

Yes

Is the study design appropriate and is the work technically sound?

Partly

Are the conclusions drawn adequately supported by the results?

Yes

Are sufficient details of methods and analysis provided to allow replication by others?

Yes

Reviewer Expertise:

Exposure to pesticides and health effects

I confirm that I have read this submission and believe that I have an appropriate level of expertise to confirm that it is of an acceptable scientific standard, however I have significant reservations, as outlined above.

# Response

**Published:** 2021-11-24

**Authors:** Chudchawal Juntarawijit

**Affiliations:** Naresuan University, Thailand

**Comment**

 This study used questionnaire-based pesticide exposure data of 20,295 farmers to determine the association between pesticide use and obesity in Thailand. With the large-scale cross-sectional epidemiological data, the study determined that regional obesity in farmers could be associated with pesticide use. The study displayed novelty and value in public health on Thailand regional health. However, I thought there were several questions in this manuscript. Therefore, I thought major revision was necessary. The detailed review points were listed below.

**Response**

 Thank you very much for your time and effort to review this manuscript and for providing valuable suggestions.

**Comment**

 1. In the abstract, the authors declared that they collected the long-term pesticide exposure data. However, I thought the term ‘long-term’ should be considered carefully as the questionnaire did not collect the frequencies of pesticides use (only yes or no). Also, the nature of the cross-sectional study (without follow-up) could poorly support the conclusion.

**Response**

 The term ‘ *long-term use*’ has been replaced with ‘ *historical use*’.

 In this study, actually, we also collected data on the frequency and duration of pesticides use and calculated cumulative days of exposure. Unfortunately, there was only a small number of cases with obesity, therefore the association between quartile days of exposure and obesity could not be performed.

**Comment**

 2. In the farmer population, pesticide-attributed obesity may be associated with the living conditions or geographical location of these individuals. Therefore, I thought it would be helpful if the authors provided the related data of these individuals.

**Response**

 In this survey, we collected data from respondents who are farmers living in a rural area of six districts but found obesity cases in five districts (Table 1).

 For socioeconomic data, only the district location of participants, and their education, and monthly income were collected.  However, these factors were not included in the model because of several reasons. For district location, most of the participants were farmers from rural areas. Moreover, the district was closely related to pesticide use and will cause multicollinearity problems, if included in the model (Bhandari, 2020). For income, there was no relationship with obesity in middle-income countries, e.g. Thailand (Ameye and Swinnen, 2019).  For education, previous research found a higher risk of obesity to be inversely associated with education (Anyanwu, 2010). However, in this study more obesity was found among those with higher education (Table 1). In addition, when these two variables were included in the model, not many effects on OR were found. For example, for molluscicide, the OR was changed from 2.28 (1.48-3.64) to 2.37 (1.48-3.79) when added education and to 2.39 (1.49-3.83) when added income (Table 2-1). For baseline diseases, most of them should be considered as the so-called ‘downstream outcome’ closely related to obesity and thus, should not be included in the model (DVL, 2020).

 More demographic information has been added to Table 1.

**Comment**

 3. Methods: Since there is a great difference between genders on obesity diagnosis, I recommended the authors perform stratified analysis to determine the gender-specific associations.

**Response**

 Thank you for bringing up a good point. Though, there is a great difference between genders on obesity diagnosis, the prevalence of obesity between gender was not different (p=0.173) (Table 1). In addition, we already added gender as a covariate in the analysis of the adjusted odds ratio as shown in Table 2 and Table 3. Moreover, the number of subjects with obesity diagnosed was too small to do stratification.

**Comment**

 4. Results: The authors checked the relations between pesticide exposure and obesity. Is there any interaction between the different pesticides?

**Response**

 That is a good point. Yes, we also believed that there might be some interaction between the different pesticides. However, due to limitations on small cases, the problem could not be further investigated. The issue has been added to the discussion section.

**Comment**

 5. Results: The authors should clarify the demographic characteristics in detail. 

**Response**

 The results and Table 1 have been revised.

**Comment**

 6. Results: It will be helpful to display the C-index of each model to validate the reliability.

**Response**

 According to Austin and Steyerberg (2012), there are three primary reasons to develop a logistic regression model. The first one is to determine the independent predictors of a binary outcome. The second one is to define the association between a predictive variable and the outcome after adjusting for confounding factors. The third reason is to predict the probability of the occurrence of a binary outcome given a specific vector of covariates. The third reason often occurs in biomedical research, where researchers are interested in predicting the prognosis of individual patients. And the C-statistic is an indicator of assessing the prediction of the logistic regression model. However, the main objective was to find the association between obesity and pesticide exposure.

**Comment**

 7. Discussion: I noticed that the OR for pesticides is from 1.75 to 8.37, while the OR over 3 may be overestimated and lead to unnecessary panic for public health. I thought the authors should discuss their results more carefully.

**Response**

 Yes, we agree with you and the statement ‘ *Some results were with high OR and wide confidence intervals, and thus should be interpreted with caution.*’ has been added to the study limitation section.

**Comment**

 8. Discussion: I thought the authors should declare the nature of the cross-sectional study and tell the readers that the study was limited in explaining the causality.

**Response**

 Thank you for your suggestion. The following statements have been added to the study limitation:

*‘There were several limitations to the study that need to be mentioned. By using a cross-sectional design, the study was limited in explaining the causality since both disease and exposure data were examined at the same time.*’

**Comment**

 9. Discussion: The strengthen part only replicates the conclusion.

**Response**

 The statement has been deleted.

**Comment**

 10. One of the most limited parts of this study was the lack of dose or level data of pesticides use, which may cause serious bias. The authors should make a discussion about this.

**Response**

 Pesticide dose is a good marker for short-term pesticide exposure. However, it was costly and may not be suitable for a large survey study. The questionnaire method is probably the best alternative to collect data on long-term pesticide exposure. The method is commonly used in epidemiological studies e.g., Agricultural Health Study in the United State. For studies using a large group of participants, the method has been proved to be useful without any serious bias (Coble et al., 2011) (Hoppin et al., 2002). The limitation on the issue was further discussed as suggested.

**Comment**

 11. The authors should explain the reliability and validity of their questionnaire as there may be information bias that exists with the questionnaire method.

**Response**

 The limitation of questionnaire use was further discussed. As mentioned before, the questionnaire method might be the only option for the study of long-term exposure to pesticides among a large population. Since the technique was used in both case and control groups, information bias if occur might be non-differential.

**Comment**

 12. I recommended the authors list the data on body measure index or waist circumstances in Table 1.

**Response**

 In this study, data on BMI and WC were not collected as most previous studies did. The study was designed to be a descriptive cross-sectional study to explore the prevalence of medical-diagnosed obesity using ICD-10 records and to identify its associated risk factors. The usefulness and limitation of the ICD-10 data were already discussed in the manuscript.  

**Comment**

 13. Waist circumferences and baseline diseases should be adjusted as covariates.

**Response**

 As mentioned before, we did not have waist circumference data since the study used a medical diagnosis of obesity (ICD-10), which is also based on BMI and waist circumferences data.

 For baseline diseases, they should be considered as a so-called ‘downstream outcome’ variable. According to Dan VanLunen (2020), this type of variable should not be used as a covariate.

**Comment**

 14. According to the previous literature, there is a great difference between populations in different age groups. Therefore, I thought age-specific associations between pesticides use and obesity should be considered.

**Response**

 We agree that age-specific association could improve study quality. This study uses a different study design from those in the literature. In this study, we use a descriptive cross-sectional design, whereas, most of the literature uses analytical cross-sectional with a control group. The analytical study might be better in control of confounding variables but not suitable for a large-scale study.

 In this study, the age-specific association was also not possible because the number of cases with obesity was small.  

**Comment**

 15. As the authors declared, the limited data on dietary patterns, physical activities, and genetic variants may lead to potential bias. However, I thought the authors should be concerned about whether their adjusted models could explain the variance.

**Response**

 Yes, we agree. More information has been added to the study limitation as follows:

 ' *Data on other risk factors, such as diet, exercise, or genetics were not collected. These confounding factors might have a different impact on the results. However, the problem may not have much effect on the study results since the case and control groups were from the same community and had the same occupation.*'

**Comment**

 16. Could the authors list the blood pressure, glucose, triglyceride, and HDL value in the tables? Also, I think adjusting them in the model should be considered.

**Response**

 As mentioned before, the study was designed as a simple descriptive cross-sectional survey to try to find a prevalence of obesity using ICD-10 data.

 Among the two types of a cross-sectional study, descriptive and analytical, each one has its own strength and limitations. The descriptive studies, which could be conducted in a large-scale study, are often used for prevalence studies. For the analytical study, a study with a control group, often found in a medical study, and has better control for confounding.   

**Comment**

 17. The effects of pesticides exposure may have co-exposure patterns, the authors should notice that.

**Response**

 Thank you for raising this good point. Yes, co-exposure was an interesting issue. However, with the study design and the small number of cases, this study could not evaluate the problem. The issue has been added in the discussion as a study limitation.

**References**

 Anyanwu, G. E., Ekezie, J., Danborno, B., & Ugochukwu, A. I. (2010). Impact of education on obesity and blood pressure in developing countries: A study on the Ibos of Nigeria. North American journal of medical sciences, 2(7), 320–324. https://doi.org/10.4297/najms.2010.2320

 Austin, P.C., and Steyerberg, E.W. (2012). Interpreting the concordance statistic of a logistic regression model: relation to the variance and odds ratio of a continuous explanatory variable. BMC Med Res Methodol 12 (82). https://doi.org/10.1186/1471-2288-12-82

 Bhandari A. (2020). What is multicollinearity? Here’s everything you need to know. Analytics Vidhya. Available: Multicollinearity | Detecting Multicollinearity with VIF (analyticsvidhya.com)

 Coble et al., (2011). An updated algorithm for estimation of pesticide exposure intensity in the agricultural health study, Int. J. Environ. Res. Public Health; 8(12), 4608–4622.  doi: 10.3390/ijerph8124608.

 Dan VanLunen (DVL). (2020). Get a Grip! When to Add Covariates in a Linear Regression. A Guide to Accurately and Precisely Measuring Effects! Available: https://towardsdatascience.com/get-a-grip-when-to-add-covariates-in-a-linear-regression-f6a5a47930e5

 Hannah Ameye and Johan Swinnen, (2019). Obesity, income and gender: The changing global relationship, Global Food Security, 23, 267-281. https://doi.org/10.1016/j.gfs.2019.09.003.

 Hoppin, F. Yucel, M. Dosemeci, and D. P. Sandler, (2002). Accuracy of self-reported pesticide use duration information from licensed pesticide applicators in the Agricultural Health Study, J. Expo. Anal. Environ. Epidemiol.; 12(5), 313–318.  doi: 10.1038/sj.jea.7500232.

# Response

**Published:** 2021-11-24

**Authors:** Chudchawal Juntarawijit

**Affiliations:** Naresuan University, Thailand

**Table 2-2  OR after adding more variables to the model.**

 Pesticide: Molluscicide

 OR (as shown in Table2)   3.36 (2.13-5.31)

 OR (after add baseline diseases)   3.30 (2.08-5.22)

 OR (add district location)   2.28 (1.43-3.64)

 OR (add education)   2.37 (1.48-3.79)

 OR (add income)   2.39 (1.49-3.83)
